# Long-Term Outcomes of IgG4-Related Ophthalmic Disease in a Chinese IgG4-Related Disease Cohort

**DOI:** 10.3389/fmed.2021.784520

**Published:** 2021-12-17

**Authors:** Linyang Gan, Xuan Luo, Yunyun Fei, Linyi Peng, Jiaxin Zhou, Jieqiong Li, Hui Lu, Zheng Liu, Panpan Zhang, Xiaowei Liu, Wen Zhang

**Affiliations:** ^1^Department of Ophthalmology, Peking Union Medical College Hospital, Chinese Academy of Medical Sciences and Peking Union Medical College, Beijing, China; ^2^Department of Rheumatology, National Clinical Research Center for Dermatologic and Immunologic Diseases (NCRC-DID), Peking Union Medical College Hospital, Chinese Academy of Medical Science and Peking Union Medical College, Beijing, China; ^3^Department of Rheumatology and Immunology, The First Affiliated Hospital of Zhengzhou University, Zhengzhou, China

**Keywords:** IgG4-related ophthalmic disease, corticosteroids, immunosuppressant, relapse, risk factor

## Abstract

**Purpose:** To define the treatment response and long-term outcomes of a large IgG4-related ophthalmic disease (IgG4-ROD) cohort.

**Methods:** A total of 132 patients with a minimum follow-up of 1 year were included in this study. Demographic, clinical, and laboratory data were collected. Treatment response was assessed by the IgG4-RD responder index (IgG4-RD RI). Risk factors for relapse were analyzed with the multivariate Cox regression analysis.

**Results:** The median follow-up time was 39 months. Lacrimal gland involvement was detected in 87.9% of cases. Extraocular muscles, the trigeminal nerve, and other soft tissue were affected in 25.8, 6.1, and 18.2% of patients. The relapse rate of watchful waiting, glucocorticoid monotherapy, immunosuppressant monotherapy, and combination therapy was 50.0, 51.7, 50.0, and 26.7% (*p* = 0.038), respectively. The combination therapy group exhibited shorter glucocorticoids therapy duration (36 vs. 48 months, *p* = 0.009) and maintenance period (24 vs. 42 months, *p* = 0.003). At the 6th month, the median IgG4-RD RI declined from 12 to 1 and 105 (79.5%) patients achieved complete response (CR). Relapse occurred in 49 (37.1%) patients. The multivariate Cox regression analysis exhibited that CR at the 6th month was an independent protective factor for relapse. Patients with multiple ocular lesions suffered from a higher risk of relapse. No patient had severe adverse reactions to the treatment in this study.

**Conclusion:** Relapse was common in patients with IgG4-ROD. Patients receiving combination therapy showed a lower relapse rate and a shorter glucocorticoids therapy period. The presence of multiple ocular lesions was associated with a higher risk of relapse. CR at the 6th month might be a predictor for a better prognosis in IgG4-ROD. Thus, a more aggressive regimen should be prescribed for patients with a poor initial response.

## Introduction

IgG4-related disease (IgG4-RD) is a recently recognized systemic inflammatory condition, which is frequently misdiagnosed as a neoplastic, inflammatory, or infectious condition (1). Involvement of ophthalmic structures, including the lacrimal gland, extraocular muscles (EOMs), branches of the trigeminal nerve, and orbital fat, is quite common in the context of Mikulicz's disease, idiopathic orbital inflammation, and orbital benign lymphoid hyperplasia before the recognition of IgG4-related ophthalmic disease (IgG4-ROD). Ophthalmic involvement of IgG4-RD was reported to be associated with higher risk of relapse (2, 3).

Thus far, because of the rarity of disease, most publications concern small case series. Larger cohorts of IgG4-ROD are needed to better understand its treatment outcomes and to provide a higher level of evidence around management of patients. The only study on a large cohort of IgG4-ROD was published recently (4). However, it did not report details of therapy and the exact relapse rate. It is still difficult to establish the optimal treatment strategy for IgG4-ROD.

In this study, we describe the demographic features, clinical manifestations, laboratory results, anatomic locations of orbital lesions, treatment regimen, and relapse data of a large Chinese cohort of IgG4-ROD. The purpose of this study is to define the treatment response and long-term outcomes for IgG4-ROD.

## Methods

In this study, 132 patients with IgG4-ROD from an IgG4-RD cohort were analyzed at Peking Union Medical College Hospital from March 2011 to March 2021 (ClinicalTrials.gov ID: NCT01670695). All the 132 patients fulfilled the 2019 American College of Rheumatology/European League Against Rheumatism (ACR/EULAR) classification criteria for IgG4-RD (5, 6). Patients were included in our analysis, if they had at least 1 ocular structure involved and a minimum follow-up time of 1 year. Patients with infectious diseases, other rheumatic diseases, malignancies, or conditions that could mimic IgG4-RD were excluded. This study was approved by the Medical Ethics Committee of Peking Union Medical College Hospital.

Ophthalmic involvement was identified both clinically and radiologically. Anatomic locations of ocular lesions were determined by orbital CT or MRI scans including the lacrimal glands, the trigeminal nerve, and EOM as well as orbital fat and eyelids. Extraophthalmic organ involvement was evaluated by manifestations, laboratory tests, and imaging studies of the thorax and/or abdomen and/or pelvic region by ultrasound (US), CT, MRI, and/or PET/CT.

Clinical records of patients, including demographics, disease duration, serological results, organ involvement, and disease activity at baseline, were collected. Patients were followed at 1 month, 3 months, and then approximately every 3 months for at least 12 months. During follow-up visits, laboratory tests were repeated regularly; imaging studies were performed according to clinical need.

A total of 4 types of initial therapy were administered: glucocorticoids (GCs) monotherapy, GCs and immunosuppressant (IMS) combination therapy, IMS monotherapy, and watchful waiting. GCs therapy included 2 stages: (1) GCs tapering period: the standard induction dose of oral prednisone was 0.6–1.0 mg/kg/day in the 1st month and was gradually decreased by 5 mg per 1 or 2 weeks to the maintenance dosage and (2) the prednisone dose was then maintained at ≤ 10 mg/day. For patients with autoimmune pancreatitis, sclerosing cholangitis, retroperitoneal fibrosis, or severe impairment of other internal organs, an IMS was administered in initial therapy regimens. If patients did not respond well to GCs therapy alone during follow-up, an IMS was added to the GCs therapy. For patients with mild disease and no internal organ involvement, we tried to avoid GCs and tended to give them IMS monotherapy. IMSs used in this study included methotrexate (MTX), cyclophosphamide (CTX), cyclosporine A (CsA), mycophenolate mofetil (MMF), leflunomide (LEF), azathioprine (AZA), hydroxychloroquine (HCQ), and iguratimod (IGU). For patients who could not withstand the potential adverse reactions of GCs and IMSs, rituximab treatment was an alternative choice. Although the therapy strategy was adjusted according to treatment response, adverse reactions, and relapse, when conducting statistical analysis, patients were categorized according to their initial therapeutic regimens.

Disease activity was assessed by the IgG4-RD responder index (IgG4-RD RI) at each visit. The main outcome was treatment response at the 6th month. An IgG4-RD RI score of ≥ 3 was used to identify patients with active disease. Clinical response was classified into 3 types (7): complete response (CR), partial response (PR), and no change. IgG4-RD RI scores < 3 and decreasing to ≥ 2 were defined as CR and IgG4-RD RI scores still ≥ 3 but decreasing to ≥ 2 were defined as PR. If IgG4-RD RI score of patients was 3 points at the beginning, PR was considered as a 1 point decrease after therapy. Patients without apparent changes in clinical manifestations as well as IgG4-RD RI scores of < 2, were considered to have no change (7). Relapse was defined as the recurrence of disease-related symptoms, swelling of organs, and worsening of imaging studies, with or without re-elevation of the serum IgG4 level. At the time of relapse, systemic corticosteroid treatment with or without IMSs was restarted. Adverse reactions to the therapy were recorded during follow-up visits.

Statistical analysis was performed using the SPSS (version 20; IBM Corporation, Armonk, New York, USA). The normality of distribution was confirmed with the Kolmogorov–Smirnov test. Continuous non-normally distributed data were presented as median (1st quartile and 3rd quartile) and compared by non-parametric test. The Bonferroni correction was performed for multiple comparisons. Categorical variables were analyzed by the Fisher's exact test or the chi-squared test. The Kaplan–Meier survival curve was used and a log-rank test was performed to compare relapse-free survival. Variables with a *p* < 0.1 in the univariate analysis and the predictors of relapse suggested in past reports were included in the multivariate Cox regression analysis with a forward selection method (likelihood ratio test) to estimate the hazard ratio (HR) of relapse. *p* < 0.05 was considered as statistically significant.

## Results

### Baseline Characteristics of Patients With IgG4-ROD

As shown in Table 1, 132 patients fulfilled our inclusion criteria, with a minimum follow-up time of 1 year (median follow-up time 39 months, ranging from 12 to 108 months). The median age was 52 years. No significant male predominance was detected in our cases. Initial manifestations occurred at 48.5 [interquartile range (IQR): 39.5–56] years. Median time from onset to diagnosis was 24 (12, 60) months. Extraophthalmic involvement was noted in most patients; the salivary gland was the most commonly affected site (74.2%), followed by the paranasal sinuses (59.8%) and lymph nodes (47.0%). The baseline IgG4-RD RI of the watchful waiting group was lower than that of the GCs group (*p* = 0.002) and the combination group (*p* < 0.001) and the IgG4-RD RI of the IMS monotherapy group was also lower than that of the combination group (*p* = 0.006). Most demographic features and baseline clinical characteristics were comparable among the 4 treatment groups, except for the proportion of salivary gland, lung, and lymph node involvement (Table 1).

**Table 1:** Demographic features and baseline clinical characteristics of patients with IgG4-ROD.

**Characteristics**	**All (*n* = 132)**	**Initial therapy**				
		**Watchful waiting**	**GC**	**GC + IMS**	**IMS**	***p*-value**
		**(*n* = 10)**	**(*n* = 29)**	**(*n* = 75)**	**(*n* = 18)**	
Male sex, n (%)	76 (57.6%)	1 (10%)	19 (65.5%)	47 (62.7)	9 (50%)	
Age (years), median (IQR)	52 (44.25, 59)	53 (41, 63.5)	49 (40, 58.5)	53 (45, 59)	52 (46.75, 56.25)	0.761
Age of onset (years), median (IQR)	48.5 (39.5, 56)	51 (39.56, 61.75)	47 (36, 56.5)	49 (41, 56)	47 (39, 52)	0.565
Time to diagnosis (months), median (IQR)	24 (12, 60)	18 (5.75, 32.25)	24 (12, 66)	24 (12, 48)	42 (12, 102)	0.172
Score of ACR/EULAR classification criteria, median (IQR)	30.5 (25, 39)	25 (20, 28.25)	29 (24, 40)	31 (25, 43)	33.5 (27.25, 38.25)	0.015
IgG4-RD RI, median (IQR)	12 (9, 15)	6 (3, 9.75)	12 (9, 18)	14 (12, 18)	9 (6, 12)	<0.001
**Symptoms at disease onset, n (%)**						
Nausea and vomiting	4 (3.0%)	0 (0.0%)	2 (6.9%)	2 (2.7%)	0 (0.0%)	0.517
Parotid gland swelling	20 (15.2%)	1 (10.0%)	6 (20.7%)	12 (16.0%)	1 (5.6%)	0.612
Submandibular gland swelling	69 (52.3%)	3 (30.0%)	20 (69.0%)	38 (50.7%)	8 (44.4%)	0.126
Lymphadenopathy	34 (25.8%)	0 (0.0%)	13 (44.8%)	19 (25.3%)	2 (11.1%)	0.011
Abdominal pain	4 (3.0%)	0 (0.0%)	2 (6.9%)	2 (2.7%)	0 (0.0%)	0.517
Jaundice	4 (3.0%)	0 (0.0%)	3 (10.3%)	1 (1.3%)	0 (0.0%)	0.094
Nasal congestion	42 (31.8%)	3 (30%)	12 (41.4%)	25 (33.3%)	2 (11.1%)	0.167
Cough	14 (10.4%)	0 (0.0%)	4 (13.8%)	10 (13.3%)	0 (0.0%)	0.310
Low back pain	4 (3.0%)	1 (10.0%)	2 (6.9%)	1 (1.3%)	0 (0.0%)	0.119
Allergy history, *n* (%)	85 (64.4%)	5 (50.0%)	19 (65.5%)	49 (65.3%)	12 (66.7%)	0.815
Number of organs involved, n (%)						<0.001
1–2	27 (20.5%)	6 (60.0%)	6 (20.7%)	6 (8.0%)	9 (50.0%)	
3–4	52 (39.4%)	3 (30.0%)	9 (31.0%)	34 (45.3%)	6 (33.3%)	
≥5	53 (40.2%)	1 (10.0%)	14 (48.3%)	35 (46.7%)	3 (16.7%)	
**Organ involvement, n (%)**						
Salivary gland	98 (74.2%)	4 (40.0%)	25 (86.2%)	58 (77.3%)	11 (61.1%)	0.019
Pancreas	24 (18.2%)	0 (0.0%)	6 (20.7%)	16 (21.3%)	2 (11.1%)	0.400
Biliary system	10 (7.9%)	0 (0.0%)	4 (13.8%)	6 (8.0%)	0 (0.0%)	0.395
Retroperitoneal fibrosis	10 (7.6%)	0 (0.0%)	1 (3.4%)	8 (10.7%)	1 (5.6%)	0.660
Lung	44 (33.3%)	1 (10.0%)	8 (27.6%)	32 (42.7%)	3 (16.7%)	0.048
Kidney	20 (15.2%)	1 (10.0%)	6 (20.7%)	13 (17.3%)	0 (0.0%)	0.198
Lymph nodes	62 (47.0%)	2 (20.0%)	19 (65.5%)	35 (46.7%)	6 (33.3%)	0.043
Paranasal sinuses	79 (59.8%)	6 (60.0%)	15 (51.7%)	45 (60.0%)	13 (72.2%)	0.601
Thyroid gland	4 (3.0%)	0 (0.0%)	2 (6.9%)	2 (2.7%)	0 (0.0%)	0.517

*IgG4-ROD, IgG4-related ophthalmic disease; IQR, interquartile range*.

Lacrimal gland involvement was detected in 116 (87.9%) cases (Table 2). EOMs, soft tissue, and branches of the trigeminal nerve were affected in 34 (25.8%), 24 (18.2%), and 8 (6.1%) patients, respectively. A total of 41 patients (31.1%) had multiple types of lesions.

**Table 2:** Anatomic locations of ocular lesions.

	**All (n = 132)**	**Initial therapy**				
		**Watchful waiting**	**GC**	**GC + IMS**	**IMS**	***p*-value**
		**(*n* = 10)**	**(*n* = 29)**	**(*n* = 75)**	**(*n* = 18)**	
Lacrimal gland, *n* (%)	116 (87.9%)	10 (100.0%)	25 (86.2%)	65 (86.7%)	16 (88.9%)	0.795
Extraocular muscle, *n* (%)	34 (25.8%)	0 (0.0%)	5 (17.2%)	21 (28.0%)	8 (44.4%)	0.041
Trigeminal nerve, *n* (%)	8 (6.1%)	1 (10.0%)	2 (6.9%)	5 (6.7%)	0 (0.0%)	0.607
Other soft tissues, *n* (%)	24 (18.2%)	2 (20.0%)	6 (20.7%)	12 (16.0%)	4 (22.2%)	0.835
Bilateral involvement, *n* (%)	124 (93.9%)	8 (80.0%)	28 (96.6%)	71 (94.7%)	17 (94.4%)	0.247
Multiple lesions, n (%)	41 (31.1%)	3 (30.0%)	9 (31.0%)	21 (28.0%)	8 (44.4%)	0.631

Laboratory findings were compared. The median IgG4 level was 13.5 g/l and 96.2% patients had elevated IgG4 levels. Immunoglobulin E (IgE) elevation was detected in 92.3% of patients. The combination therapy groups showed higher serum IgG4 levels (*p* = 0.005) and serum IgG4/IgG ratios (*p* = 0.002) than the watchful waiting group. Other findings, including IgG, total IgE levels, C-reactive protein (CRP), and Erythrocyte sedimentation rate (ESR), were comparable among the 4 treatment groups.

Biopsy was performed in 104 (78.8%) cases. A dense lymphoplasmacytic infiltration was the main feature in 94.2% cases, with immunohistochemistry for IgG4-positive plasma cell count > 10/High-power field (HPF) and 81% specimens showing an IgG4/IgG ratio > 40%. The IgG4/IgG ratio was not significantly different between the 2 relapsed and non-relapsed groups (*p* = 0.082). Fibrosis was mentioned in 78.8% of biopsies including a storiform pattern.

### Treatment and Outcomes of Patients With IgG4-ROD

As shown in Table 3, 104 patients received GC-based initial therapy and 18 patients were prescribed it during follow-up. Initial GCs dosage was 40 (30, 40) mg prednisone equivalent; this was similar between the GC monotherapy and combination therapy groups (*p* = 0.928). Median GC therapy duration was recorded up to 33 months. Low-dosage GC maintenance therapy (10 mg/day and lower) was administered to 95 (91.3%) patients in the GC and GC + IMS groups, with a median maintenance dose of 7.5 mg of prednisone equivalent and a median maintenance period of 30 months. GCs were discontinued successfully in 13 (10.6%) patients eventually. The combination group exhibited a shorter GC therapy duration (*p* = 0.009) and maintenance period (*p* = 0.003).

**Table 3:** Treatment and outcomes of patients with IgG4-ROD.

	**Initial therapy**				
	**Watchful waiting**	**GC**	**GC + IMS**	**IMS**	***p*-value**
	**(*n* = 10)**	**(*n* = 29)**	**(*n* = 75)**	**(*n* = 18)**	
**Therapy adjusted during follow-up**					
GC added	2 (20.0%)			11 (61.1%)	
GC+IMS added	5 (50.0%)				
IMS added	1 (10.0%)	26 (89.7%)			
Initial GC dose		40 (35, 40)	40 (30, 40)		0.928
GC therapy period		48 (34.5, 75)	36 (24,51)		0.009
Maintenance therapy		28 (96.6%)	67 (89.3 %)		0.240
Maintenance dose		7.5 (5.625, 8.4375)	6.875 (5, 10)		0.208
Maintenance period		42 (27.75, 53)	24 (18, 36)		0.003
GC discontinuation		1 (3.4%)	11 (14.7%)		0.206
Number of IMS			2 (1, 2)	2 (1.75, 3)	0.159
IMS switches			1 (0, 1)	1 (0, 1.25)	0.656
IMS withdrawal			13 (17.3%)	1 (5.6%)	0.375
RI change	4.5 (2.25, 9.25)	12 (6, 16)	12 (10, 14)	7 (4, 11)	<0.001
RI at 6^th^ month	1.5 (0.25, 2)	2 (1, 3)	1(1, 2)	2 (1, 2)	0.253
CR at 6^th^ month	7 (70.0%)	19 (65.5%)	63 (84.0%)	16 (88.9%)	0.116
Relapse	5 (50.0%)	15 (51.7%)	20 (26.7%)	9 (50.0%)	0.038

*GC, glucocorticoids; IMS, immunosuppressants; RI, responder index; CR, complete response*.

Immunosuppressants were given to 93 (70.5%) patients as an initial treatment. During follow-up, an additional 32 (24.2%) patients received IMSs as add-on therapy. Most patients received more than 1 class of IMS during the follow-up (ranging from 2 to 4 classes) due to de-escalation therapy, adverse reactions, or economic concerns. As an initial therapy, CTX was the most commonly prescribed IMS (*n* = 31), followed by MTX (*n* = 28) and MMF (*n* = 22). During follow-up, the rank switched to MTX (*n* = 32), CTX (*n* = 29), and MMF (*n* = 25). Only 4 patients were able to stop all the medications at the end of follow-up. Rituximab was used in 3 cases. Case 1 received 2 doses of rituximab (600 mg and 500 mg) after the 2nd relapse, combined with GCs and CTX. No further relapse was observed at the 4-year follow-up. Case 2 received a 4-weekly 500 mg rituximab infusions and GCs as an initial treatment; he achieved complete remission for 4 years. A 4-weekly 500 mg rituximab infusions were administered as monotherapy in case 3. Disease activity was not well-controlled and a GC was added.

Treatment strategies were adjusted during follow-up. As shown in Table 3, 8 patients in watchful waiting received GCs or IMSs because of worsening or relapse. Similarly, 26 patients from the GC monotherapy group and 11 patients from the IMS monotherapy group were switched to the combination therapy group.

**Figure 1:**
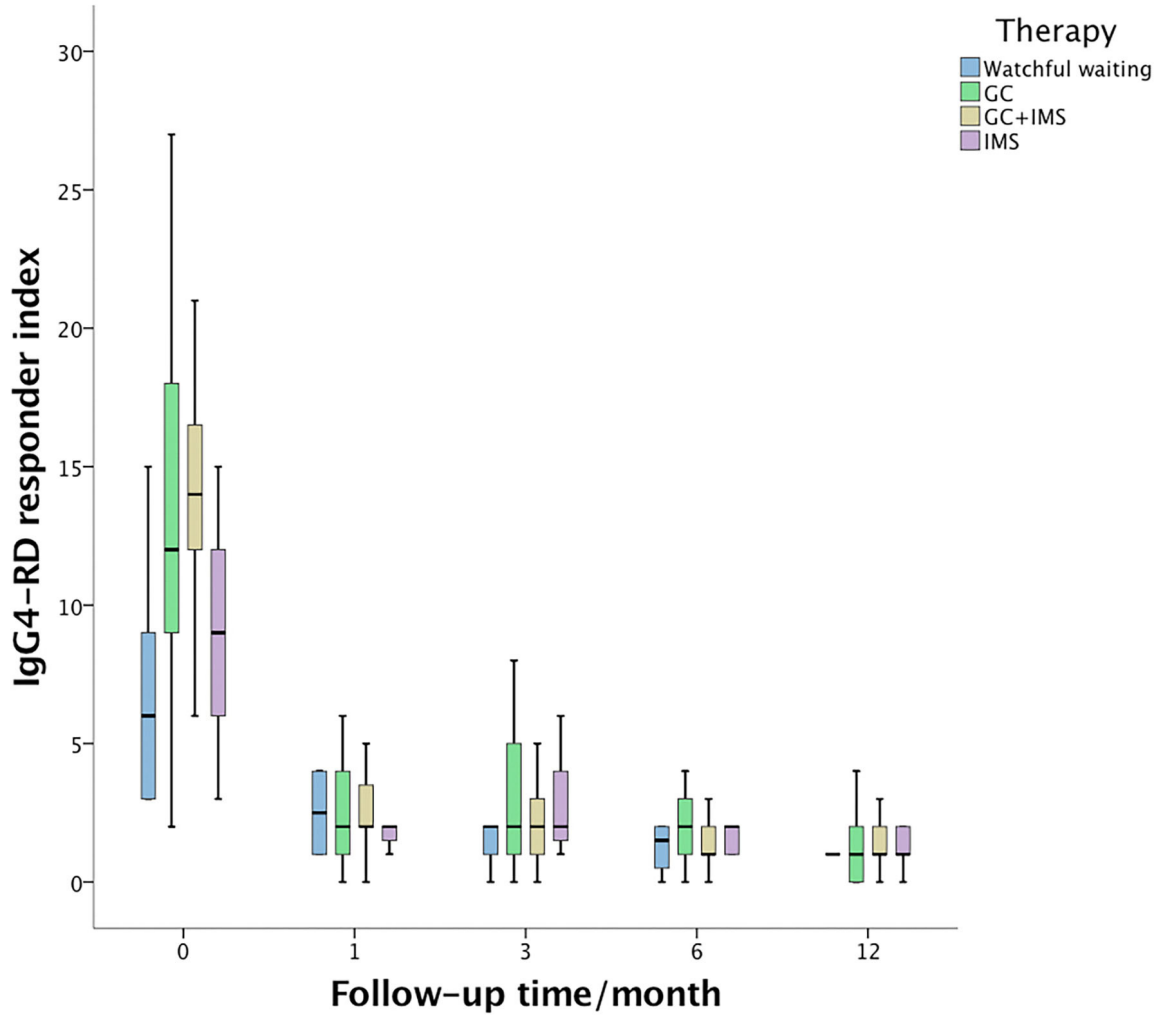
IgG4-related disease responder index (IgG4-RD RI) changes during 1st year of follow-up. The baseline IgG4-RD RI of the combination therapy group (yellow box) was higher than that of the watchful waiting group (blue box, *p* < 0.001) and the immunosuppressant monotherapy group (purple box, *p* = 0.006). Then, IgG4-RD RI dropped dramatically during follow-up and showed no statistical significance among the 4 groups.

At the 6th month, the median IgG4-RD RI declined dramatically even in the watchful waiting group (Figure 1). The combination therapy group exhibited larger IgG4-RD RI decline than the watchful waiting group (*p* = 0.002) and IMS monotherapy group (*p* = 0.001). A total of 105 (79.5%) patients achieved CR at the 6th month. CR rates were similar among 4 groups (*p* = 0.116). Patients who achieved CR showed similar GCs doses to patients without CR from the baseline (*p* = 0.886), the 1st month (*p* = 0.407), the 3rd month (*p* = 0.337), and the 6th month (*p* = 0.214). However, the ratios of initial IMS treatment were higher in the CR group (75.2 vs. 47.6%, *p* = 0.011).

The median IgG4 level dropped to 3.80 (1.86, 6.39) g/l at the 6th month, with 84 (63.6%) cases still exceeding normal level. At the end of follow-up, 115 (87.1%) patients had elevated serum IgG4.

### Associated Factors for Relapse

**Figure 2:**
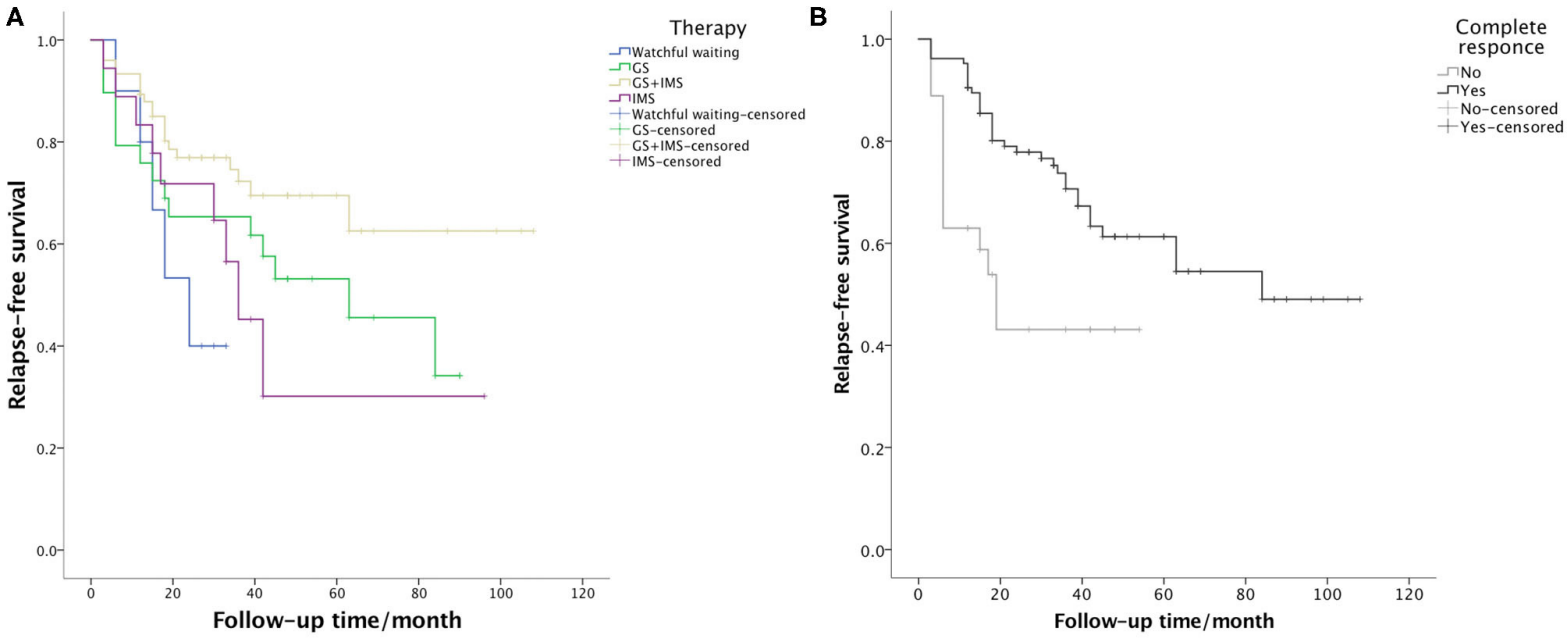
Survival curve presenting the relapse-free survival of **(A)** the 4 therapy groups (*p* = 0.059) and **(B)** patients who achieved and did not achieve CR at the 6th month (*p* = 0.002).

Relapse occurred in 49 (37.1%) patients. The median time of relapse was 15 months (from 3 to 84 months). A total of 9 patients experienced 2 relapses and 3 patients relapsed 3 times. Relapse after discontinuing GCs was recorded in 5 patients using GC-based therapy. In 35 patients, relapse occurred during GC tapering and maintenance, usually when the GCs were under 7.5 mg/day (21/35). The most commonly relapsed organs were the lacrimal glands (31, 63.3%), followed by the lungs (8, 16.3%) and the paranasal sinuses (4, 8.2%). Serum IgG4 level increased at relapse in 36 (73.5%) patients. After relapse, the dose of GCs was increased in 21 cases. IMS dosage was adjusted in 9 cases, with 1 increasing their dose, 4 switching to another IMS, and 4 adding a new IMS. A total of 31 patients achieved CR after relapse. The relapse rate of watchful waiting, GC monotherapy, IMS monotherapy, and combination therapy was 50.0, 51.7, 50.0, and 26.7% (*p* = 0.038), respectively. The Kaplan-Meier survival analysis (Figure 2) revealed that CR at the 6th month was associated with longer relapse-free survival (*p* = 0.002). The combination therapy group exhibited longer relapse-free survival of borderline statistical significance (*p* = 0.059). As shown in Table 4, CR at the 6th month was an independent protective factor in the multivariate Cox regression analysis (*HR* = 0.401, *p* = 0.005). Patients with multiple ocular lesions suffered from a higher risk of relapse (*HR* = 2.074, *p* = 0.012). Other variables, including sex, age at onset, eosinophils, serum IgG4 level, kidney disease, therapy group, and GC maintenance therapy, were not associated with a relapse in the multivariate Cox regression analysis.

**Table 4:** The univariate analysis and the multivariate Cox regression analysis for potential risk factors associated with relapse in IgG4-ROD.

**Variables**	**Univariate analysis**		**Multivariate analysis**	
	**HR (95% CI)**	***p*-value**	**HR (95% CI)**	***p*-value**
Sex (Male)	1.545 (0.849–2.813)	0.154	-	-
Age of onset (years)	0.996 (0.975–1.017)	0.680	-	-
Time to diagnosis (months)	0.999 (0.994–1.003)	0.565	-	-
IgG4-RD RI	1.000 (0.956–1.046)	0.993	-	-
Allergy history	1.020 (0.566–1.839)	0.947	-	-
Eosinophils	1.056 (1.000–1.116)	0.052		
Serum IgG4	1.00 (1.00–1.00)	0.496	-	-
Number of organs involved	0.949 (0.811–1.111)	0.515	-	-
Organ involvement			-	-
Salivary gland	0.751 (0.403–1.400)	0.367	-	-
Pancreas	0.627 (0.282–1.397)	0.254		
Biliary system	1.213 (0.481–3.062)	0.683	-	-
Retroperitoneal fibrosis	0.515 (0.159–1.674)	0.270	-	-
Lung	0.714 (0.379–1.347)	0.298	-	-
Kidney	0.414 (0.149–1.151)	0.091	-	-
Paranasal sinuses	1.197 (0.673–2.129)	0.540	-	-
Initial therapy		0.078		
GC	1			
Watchful waiting	1.704 (0.600–4.840)	0.317		
IMS	1.260 (0.545–2.914)	0.589		
GC+IMS	0.585 (0.298–1.149)	0.120		
Initial GC dose	0.988 (0.973–1.003)	0.123	-	-
GC therapy period	0.994 (0.982–1.005)	0.267	-	-
GC maintenance	0.504 (0.249–1.021)	0.057		
CR at 6^th^ month	0.373 (0.191–0.728)	0.004	0.401 (0.212–0.756)	0.005
**Anatomic locations of ocular lesions**				
Lacrimal gland	1.099 (0.468–2.584)	0.828	-	-
EOM/CNV	1.116 (0.608–2.05)	0.723	-	-
Other soft tissues	2.237 (1.201–4.167)	0.011	-	-
Multiple lesions	2.095 (1.186–3.699)	0.011	2.074 (1.173–3.666)	0.012

*GC, glucocorticoids; IMS, immunosuppressants; RI, responder index; CR, complete response*.

*Sex, age at onset, eosinophils, serum IgG4 level, kidney disease, initial therapy, GC maintenance therapy, CR at 6th month, anatomic locations of ocular lesion, and multiple ocular lesions were included in the multivariate Cox regression analysis*.

### Adverse Reactions

Adverse reactions to the therapy are shown in Table 5. Gastrointestinal reaction, infection, GC-induced diabetes, and hypertension were the most common adverse reactions. In particular, 3 patients developed GC-induced cataracts during treatment. The IMS was withdrawn in 10 patients because of adverse reactions. No patient had severe adverse reactions—severe infection and uncontrolled renal or liver dysfunction—to the therapy. No lymphoma was observed during the course of this study.

**Table 5:** Cases with adverse events during treatment.

Infection	11
Herpes zoster	5
Pneumonia	4
Urinary tract	2
Gastrointestinal reaction	21
Diabetes mellitus	8
Hypertension	7
Liver dysfunction	3
Cataract	3
Leukopenia	2
Renal dysfunction	1
Other adverse reaction	21

## Discussion

This study focused on the treatment and long-term outcomes of IgG4-ROD in a large cohort. The aim of this study was to establish a better treatment strategy for IgG4-ROD.

Lacrimal glands were the most frequently affected ophthalmic structure in this study. Previous study has shown relapse risk varies among patients with different orbital site involvement and EOM/trigeminal nerve enlargement was a significant risk factor for relapse (8). However, the univariate analysis in this study revealed that orbital soft-tissue involvement and multiple ocular lesions were associated with relapse, while the multivariate COX regression analysis suggested that damage to more than 1 ophthalmic structure was an independent risk factor. Takahashi et al. found that involvement of 2 or more sets of lacrimal glands and/or major salivary glands was related to greater systemic disease activity (9). With regard to IgG4-ROD, other authors assumed that EOM and trigeminal nerve enlargement were secondary ocular adnexal IgG4-related lesions independent of lacrimal glands, which might be refractory to treatment (8). Thus, multiple types of ocular lesions might suggest a progressive condition of IgG4-ROD and indicate higher immune activity.

Although IgG4-ROD responds to treatment well, maintaining remission is still challenging. In this study, baseline IgG4-RD RI declined from 12 to 1 after 6 months of therapy, with the CR rate up to 79.5%. However, 37.1% of patients relapsed during follow-up and only 4 patients successfully discontinued all the medications eventually. The published relapse rate of IgG4-RD varies among different reports, ranging from 15 to 60% (10). Relapse is more common in patients with orbital involvement (2). Hong et al. reported systemic maintenance of corticosteroids over 5 months that could be suggested for initial treatment to reduce the risk of recurrence (11). When patients had fewer doses and shorter periods of initial steroid treatment, they were more likely to experience recurrence. In this study, 104 patients received GC-based initial therapy and 91.3 patients received corticosteroid maintenance therapy for a median time of 30 months. This might partially explain the relatively low relapse rate in this cohort. Moreover, the risk of relapse may also be associated with organ involvement, ethnicity, genetics, or environmental factors as well as follow-up time (2).

A systematic review of IgG4-RD showed indicators that could predict relapse included a younger age, elevated serum IgG4 levels, multiorgan involvement, a higher IgG4-RI baseline, increased eosinophilic count, and a history of relapse (10). In particular, in patients with IgG4-ROD, another study reported that when treated with GC monotherapy, higher baseline IgG4-RI, involvement of more than 5 organs, eosinophilia, and dacryoadenitis were risk factors for relapse (3). These reports were inconsistent with the results of the multivariate Cox regression analysis in this study, which detected no association between relapse risk and factors including onset age, serum IgG4 levels, multiorgan involvement, baseline IgG4-RI, and eosinophil. These disparities may be due to phenotypes of pathology among different ethnicities (10), sample size, and follow-up time for different cohorts. Some observations suggested a lower relapse rate and a higher remission rate in the combination therapy group compared with the GCs-alone group (10). In this study, the relapse rate was also relatively lower in the combination therapy group. However, the majority of patients in this study received the combination regimen; the sample size of GC and IMS monotherapy group was small. This may explain why the multivariate Cox regression analysis failed to identify the influence of therapy choices on relapse risk.

An elevated serum IgG4 level is a hallmark of IgG4-RD, but with relatively low specificity. In this study, most patients showed elevated IgG4 levels at initial estimation and did not go down to a normal level even when they achieved CR at the 6th month. Although previous reports suggested that high serum IgG4 levels before treatment were associated with relapse (11), our data exhibited similar IgG4 levels and IgG4/IgG ratios between the relapsed and non-relapsed groups. An increase in the IgG4 level was common when patients experienced relapse. It is still controversial as to whether an isolated increase of serum IgG4 concentration is a risk factor for clinical relapse. In 1 published case series, 2 out of 5 serological relapse patients turned into clinical relapse patients (7). However, since there was no such patient in this cohort, our data were insufficient and we could not draw any definitive conclusions from them.

Glucocorticoids were the first-line medication for IgG4-ROD. Patients usually responded to initial therapy well, but relapses were common (12). The initial dose of corticosteroids was suggested to be 0.6–1 mg/kg/day of prednisone equivalent for at least 2–4 weeks, followed by gradual tapering over a period of 3 to 6 months (13). One randomized controlled trial investigated treatment response of patients to high-dose (0.8–1 mg/kg) and medium-dose (0.5–0.6 mg/kg) corticosteroids (14). The medium-dose group showed a similar remission rate, but higher frequency of relapse (14). A systemic review reported that more relapses were observed when GCs were discontinued (15). Although no consensus exists about the dose required and duration of maintenance treatment, it is suggested that maintenance therapy should be continued for up to 3 years, especially for patients presenting with factors that may lead to a higher risk of relapse (1). A higher risk of flare was observed among patients receiving very low-dose maintenance therapy (<5 mg/day) (7, 16). In this study, relapsed patients showed a higher maintenance dose and lower discontinuation rate than non-relapsed patients, indicating potential resistance to treatment. However, they demonstrated no significant differences in dose and total duration of corticosteroid therapy, proportion of maintenance therapy, or maintenance duration. The multivariate Cox regression analysis suggested prompt GC treatment without watchful waiting that might be beneficial to disease control.

In this cohort, 93 patients were prescribed with at least 1 kind of IMS during follow-up. The most commonly used immunosuppressive agents were MTX, MMF, and CTX. It is believed that in combination with GCs, IMSs can reduce the relapse rate and assist with discontinuing GCs. However, little evidence exists about the exact efficacy of these drugs and most data came from retrospective studies (1). The choice of immunosuppressive agents varies among different studies. A meta-analysis of 95 patients with IgG4-ROD also reported that MTX and MMF were widely used in combination with GCs (15). AZA was another frequently used IMS in literature (2, 17–19). We did not choose AZA, as it can cause myelosuppression in Chinese patients.

A meta-analysis of 1,169 patients with IgG4-RD reported that treatment of patients with IgG4-RD with combination therapy was associated with higher remission rates and lower relapse rates (10). It was confirmed that MMF combined with steroid therapy could lead to a higher remission rate in a randomized clinical trial of IgG4-RD (7). In this cohort, the relapse rate of the combination therapy group was nearly half that of the other 3 groups. Moreover, the total GC therapy duration and maintenance period were both much shorter in the combination therapy group. Therefore, with data increasingly showing that combination therapy at the start of treatment leads to a lower relapse rate and a lower total GC dose, combination therapy has become our first choice in the recent years.

Rituximab is increasingly used in IgG4-RD. Several retrospective studies and a systematic review reported rituximab therapy led to a larger reduction in the relapse rate compared with GC and IMS therapy (1, 10, 20). In this study, 2 out of 3 patients administered with rituximab achieved long-term remission. However, rituximab is not covered by general medical insurance and the high cost limits its off-label administration. Additionally, there is no consensus on the protocols of administration. The dosage and interval between rituximab doses differ from investigator to investigator (20–23). Larger randomized controlled trials are needed to provide more evidence.

A total of 2 patients in this cohort received no medication at all during a more than 2-year follow-up. IgG4-RD RI declined from 3 to 0. Not all the manifestations of IgG4-RD require immediate treatment. “Watchful waiting” may be appropriate for patients with mild disease such as isolated mild lacrimal gland enlargement. However, these patients need to be carefully monitored and the treatment strategy should be adjusted as soon as there is evidence of deterioration.

IgG4-related ophthalmic disease responder index declined rapidly after treatment, even at the 1st month. At the 6th month, 79.5% of all the patients and 71.4% of relapsed patients achieved CR. Interestingly, the multivariate Cox regression analysis showed treatment response at the 6th month as the strongest predictive factor for relapse. In addition, we found patients who achieved CR at the 6th month had received a similar treatment regimen to those without CR in this cohort. These results suggested that patients with a poor initial response (6 months in this study) might have a worse case of IgG4-ROD and can be refractory to systemic corticosteroid treatment. Those patients should be treated more aggressively to reduce the risk of relapse.

This study had several limitations. First, it was retrospective and the results should be considered exploratory. Second, almost 60% of the patients required switches to other IMSs because of insufficient response, side effects, or economic concerns. Thus, statistical analyses of IMSs failed to draw conclusions. Third, this study was conducted at a single institution; multicenter studies with a larger sample size are needed to draw a solid conclusion.

In conclusion, we analyzed clinical features and long-term outcomes of a large IgG4-ROD cohort. Although patients responded well to initial treatment, relapse was common. Combined with GCs, IMSs could reduce the relapse rate and help with the discontinuation of GCs. Multiple ophthalmic lesions were found frequently in patients with IgG4-ROD and were associated with higher risk of relapse. Moreover, our data demonstrated that CR at the 6th month might be a strong predictor for a better prognosis in IgG4-ROD. Thus, patients with a poor initial response may need more aggressive treatment. However, a prospective clinical trial is needed to confirm this hypothesis.

## Data Availability Statement

The raw data supporting the conclusions of this article will be made available by the authors, without undue reservation.

## Ethics Statement

The studies involving human participants were reviewed and approved by the Medical Ethics Committee of Peking Union Medical College Hospital. The patients/participants provided their written informed consent to participate in this study.

## Author Contributions

LG, XLu, XLi, and WZ conceived the idea of the study and interpreted the results. LG and XLu analyzed the data. YF, LP, JZ, JL, HL, ZL, and PZ collected the data. LG wrote the paper. All authors discussed the results and revised the manuscript.

## Funding

This study was supported by the National Natural Science Foundation of China (81771757 and 82071839), the Non-profit Central Research Institute Fund of Chinese Academy of Medical Sciences (NWB20203346 and 2018PT32029), the Capital's Funds for Health Improvement and Research (No. 2020-2-4017), and the Beijing Municipal Science and Technology Commission (No. Z201100005520023).

## Conflict of Interest

The authors declare that the research was conducted in the absence of any commercial or financial relationships that could be construed as a potential conflict of interest.

## Publisher's Note

All claims expressed in this article are solely those of the authors and do not necessarily represent those of their affiliated organizations, or those of the publisher, the editors and the reviewers. Any product that may be evaluated in this article, or claim that may be made by its manufacturer, is not guaranteed or endorsed by the publisher.
